# Prevalence of bee viruses in *Apis cerana cerana* populations from different locations in the Fujian Province of China

**DOI:** 10.1002/mbo3.830

**Published:** 2019-03-18

**Authors:** Aqai Kalan Hassanyar, Shaokang Huang, Zhiguo Li, Muhammad Rizwan, Shahid Mehmood, Muhammad Fahad Raza, Muhammad Qasim, Mubasher Hussain, Songkun Su

**Affiliations:** ^1^ College of Bee Science Fujian Agriculture and Forestry University Fuzhou China; ^2^ College of Life Sciences Fujian Agriculture and Forestry University Fuzhou China; ^3^ Key Laboratory of Tropical Forest Ecology Chemical Ecology Group Xishuangbanna Tropical Botanical Garden Chinese Academy of Sciences Kunming China; ^4^ College of Plant Protection Fujian Agriculture and Forestry University Fuzhou China; ^5^ College of Horticulture Fujian Agriculture and Forestry University Fuzhou China

**Keywords:** *Apis cerana cerana*, Fujian, honeybee, prevalence, RT‐PCR, viruses

## Abstract

Prevalence of honeybee viral diseases has recently been causing major problems in the beekeeping industry, causing economic losses worldwide. Honeybees are susceptible to a variety of diseases and various pathogens. Among these pathogens, prevalence viruses, along with other factors, are seriously threatening the health of bee species. In the present study, samples were collected from 80 *Apis cerana cerana* (*A*. *c. cerana*) colonies from three different locations, Cangshan, Fuan, and Yongtai, in the Fujian Province of China. All samples were screened using the reverse transcription polymerase chain reaction (RT‐PCR) method for detection of seven honeybee viruses, namely, Chinese sacbrood virus (CSBV), deformed wing virus (DWV), Israeli acute paralysis virus (IAPV), black queen cell virus (BQCV), chronic bee paralysis virus (CBPV), acute bee paralysis virus (ABPV), and Kashmir bee virus (KBV). Our results showed that CSBV was the most prevalent as it was detected in (90%), of the samples, DWV was detected in (81.25%), and IAPV was detected in (26.25%). In contrast, insignificant prevalence results were obtained from all apiaries for BQCV, CBPV, APBV, and KBV, which were not detected in any sample. Here, we are providing the first report on the molecular detection of honeybee viruses, especially the prevalence of IAPV, from different regions in the Fujian Province of China with a high prevalence of bee viruses, on *A*. *c. cerana,* and there is great concern for the presence of honeybee viruses in the population of the native honeybee (*A*. *c*. *cerana*) in China.

## 1 INTRODUCTION

The honeybee *Apis cerana cerana* is an important bee species and economic insect and is widely maintained in China (Shi et al., 2013). Honeybees, including *A. c. cerana* species, provide a considerable economic benefit to the apicultural industry and play a vital role in the pollination of crops (Shi et al., 2013; Yang, Peng, Li, & Kadowaki, 2013).

Honeybees are suffering from a variety of diseases and infection with various pathogens, especially viruses (Zhang, Huang, Xu, Han, & Chen, 2013). Honeybee viral diseases are creating a major problem in the beekeeping industry in most regions around the globe, causing economic losses worldwide (Gisder & Genersch, 2015, 2017; Martinello et al., 2017). The number of RNA and DNA viruses that have impacted bee species as investigated by metagenomics and applied next‐generation sequencing technologies are increasing; at present, 27 honeybee viruses have been identified (Galbraith et al., 2018; McMenamin & Flenniken, 2018; Schoonvaere, Smagghe, Francis, & de Graaf, 2018). The seven most common and destructive bee viruses are sacbrood virus (SBV), deformed wing virus (DWV), black queen cell virus (BQCV), Israeli acute paralysis virus (IAPV), chronic bee paralysis virus (CBPV), acute bee paralysis virus (ABPV), and Kashmir bee virus (KBV) (Ai, Yan, & Han, 2012). Chinese sacbrood virus (CSBV) and DWV are the most widespread and distributed viruses among *A*. *c. cerana* colonies in China (Ai et al., 2012; Zhang et al., 2013). Meanwhile, in recent years, DWV, IAPV, and BQCV have also created a major problem in the beekeeping industry (Ward et al., 2007). China has more than 2 million *A*. *c. cerana* colonies, but recently, the number of colonies is declining because of the presence of bee viruses, along with some other threatening factors (Huang et al., 2017; Shan et al., 2017; Shi et al., 2013; Wang et al., 2012). The devastation of bees by viruses of the native honeybee (*A*. *c. cerana*), which are widely maintained in southern China, causes colony losses, and most of the studies focus on the European honeybee (Ai et al., 2012). Investigation and molecular research on the Chinese honeybee *A*. *c. cerana* are scarce (Shi et al., 2013).

RT‐PCR was conducted to study the prevalence and distribution of seven bee viruses in *A*. *c. cerana* colonies. Among these seven viruses, three viruses (CSBV, DWV, and IAPV) were detected with high prevalence. However, BQCV, ABPV, CBPV, and KBV were not detected in any sample. This study has provided a recent report on the distribution of bee viruses in the different geographic regions of Fujian Province and evidence of IAPV infection in *A*. *c. cerana* colonies, which has not been reported before in this species from Fujian Province, and it may be helpful for the possible control of viral epidemic diseases.

## 2 MATERIAL AND METHODS

### 2.1 Sample collection

**Figure 1:**
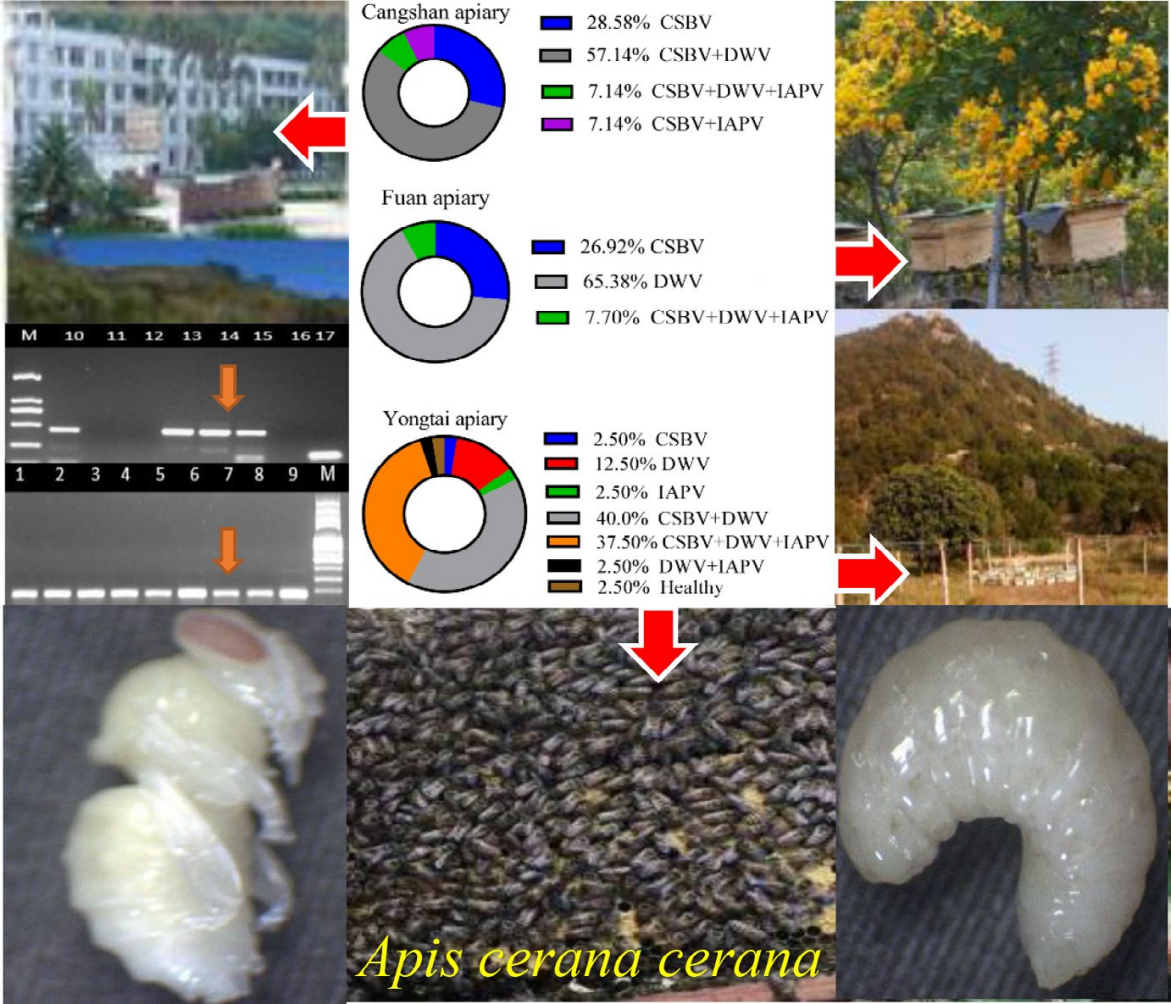
Sample collection sites from three different locations, namely, Fuan, Cangshan and Yongtai (*n* = 26, 14 and 40 colonies, respectively), and virus detection from larvae and pupae using RT‐PCR, gel electrophoresis and percentage of infected *A. c. cerana* colonies

Samples were collected from 80 *A*. *c. cerana* colonies in the Fujian Province of China during the period of October to December 2017. Samples were collected from three different regions with a large population of *A*. *c. cerana* colonies, namely, Fuan (47°15′0″N, 132°2′0″E): 26 colonies, Cangshan (°31′8″N, 119°18′58″E): 14 colonies, and Yongtai (°54′0″N, 118°56′0″E): 40 colonies (Figure 1). A total of 1,200 live samples were randomly collected (Ten 4^th^ instar larvae and five capped pupae) from every colony separately. Each apiary was randomly selected, and samples were collected from 6 colonies with an obvious symptom of a sacbrood virus infection from Fuan apiary and from 74 colonies without any exhibited symptoms of any virus infection. Samples were collected from three combs of each hive based on the frames. We selected the combs from both the side and the center of the hives. After collection from the frames, all samples were immediately transferred into a sterilized 2 ml centrifuge tube and were kept in 700 μl RNA later (Solarbio^®^ Life Science) solution according to the manufacturer's instructions. Later, larvae and pupae samples were grounded in a single tube, and 30 μl was taken from a pool of ground samples for further molecular analysis.

### 2.2 RNA extraction and cDNA synthesis

Total RNA was extracted from collected samples using a LabServ Universal RNA kit (Thermo Scientific KingFisher Flex) with magnetic beads according to the manufacturer's instructions. The concentration of RNA was determined by spectrometer using a NanoDrop ND‐2000 (Thermo Scientific). Extracted RNA was used immediately for generation of first strand cDNA to eliminate genomic DNA, certifying that only RNA existed in the final preparation. Total RNA (4 μg for a 20 μl reaction) was used to obtain cDNA. The RNA template was primed using an oligonucleotide‐deoxythymidine (oligo‐dT) primer with Transcript^®^ One‐Step gDNA Removal and cDNA Synthesis SuperMix (TransGen Biotech Beijing) according to the manufacturer's instructions. Reactions were incubated at 65°C for 5 min, 4°C for 2 min, 25°C for 10 min, 42°C for 90 min, and 85°C for 10 s, and cDNA was kept at −20°C for later use.

### 2.3 RT‐PCR assays for virus detection

An RT‐PCR was developed for better diagnosis of viral diseases by the following standard method for the accurate detection of several honeybee viruses (Yoo, Thi et al., 2012). RT‐PCR was conducted to detect seven common bee viruses (CSBV, DWV, BQCV, KBV, ABPV, IAPV, and CBPV). We screened all the samples using specific primer pairs (Table 1). Amplification of cDNA was carried out using 2X EasyTaq^®^ PCR SuperMix (TransGen Biotech Beijing). We used a total reaction volume of 15 μl in the PCR for diagnosis of each virus. For CSBV detection, PCR amplification was conducted via initial denaturation at 95°C for 1:30 min, followed by 39 cycles of 95°C for 30 s, 50°C for 30 s, and 72°C for 25 s, and finally 72°C for 1 min. For the detection of DWV, PCR amplification was conducted via an initial denaturation at 95°C for 1:30 min, followed by 39 cycles at 95°C for 15 s, 57°C for 30 s, and 72°C for 30 s, and finally 72°C for 1 min. Likewise, for IAPV detection, PCR amplification was conducted through an initial denaturation at 95°C for 1:30 min, followed by 39 cycles at 95°C for 30 s, 60°C for 30 s, and 72°C for 1 min, and finally 72°C for 2 min. CBPV, ABPV, BQCV, and KBV were not detected from any samples, so we did not mention the PCR amplification protocol here. The PCR products were analyzed by electrophoresis in a 2% 50X TBE buffer agarose gel and stained with ethidium bromide. Amplified products were visualized under UV light, and their length was compared to a standard 100‐bp molecular weight DNA ladder (Invitrogen, USA). To ensure that the PCRs were running correctly, we used an apis‐β‐actin primer pair, size: 181 bp (Table 1), for an internal positive control in all PCR virus detection assays, and for the negative control, we used double‐distilled water (ddH_2_O). We also used previously identified positive sample cDNA as a positive control for CSBV and DWV in every PCR reaction.

**Table 1:** A list of primer pairs used in RT‐PCR to detect seven bee viruses in the larvae and pupae of *Apis cerana cerana*

Primers	Sequence (5′‐3′)	Product size (bp)	Source or references
SBV‐F	GCTGAGGTAGGATATTTGCGT	824	Chen, Pettis, Collins, & Feldlaufer (2006)
SBV‐R	TCATCATCTTCACCATCCGA		
KBV‐F	GATGAACGTCGACCTATTGA	415	Thu et al. (2016)
KBV‐R	TGTGGGTTGGCTATGAGTCA		
ABPV‐F	TTATGTGTCCAGAGACTGTATCCA	900	Choe, Nguyen, Hyun et al. (2012) and Choe, Nguyen, Jin et al. (2012)
ABPV‐R	GCTCCTATTGCTCGGTTTTTCGGT		
CBPV‐F	AGTTGTCATGGTTAACAGGATACGAG	455	Singh et al. (2010)
CBPV‐R	TCTAATCTTAGCACGAAAGCCGAG		
BQCV‐F	TGGTCAGCTCCCACTACCTTAAAC	700	
BQCV‐R	GCAACAAGAAGAAACGTAAACCAC		
IAPV‐F	GGTCCAAACCTCGAAATCAA	840	
IAPV‐R	TTGGTCCGGATGTTAATGGT		
DWV‐F	CTCGTCATTTTGTCCCGACT	423	
DWV‐R	TGCAAAGATGCTGTCAAACC		
CSBV‐F	ACCTTCATCCAGTATCAGAACCAT	157	Huang et al. (2017)
CSBV‐R	ATAACCACCCGTCCCAGAG		
apis‐β‐actin‐F	AATTTTCATGGTGGATGGTGC	181	Chen, Higgins, & Feldlaufer (2005)
apis‐β‐actin‐R	AGGAATGGAAGCTTGCGGTA		

SBV, sacbrood virus; KBV, Kashmir bee virus; ABPV, acute bee paralysis virus; CBPV, chronic bee paralysis virus; BQCV, black queen cell virus; IAPV, Israeli acute paralysis virus; DWV, deformed wing virus; CSBV, Chinese sacbrood virus; and apis‐β‐actin primer.

### 2.4 Statistical analysis

Statistical analysis was carried out using SPSS version 21.0 (Inc., Chicago, Illinois, USA).

To compare the means distribution of each detected virus between different locations. Data were analyzed using one‐way ANOVA, Post Hoc Test; Tukey HSD, with *p*‐value of ≤0.05 considered significant difference. GraphPad Prism version 7.00 for Windows (GraphPad Software, California, USA) was used to draw the graphs.

## 3 RESULTS

**Figure 2:**
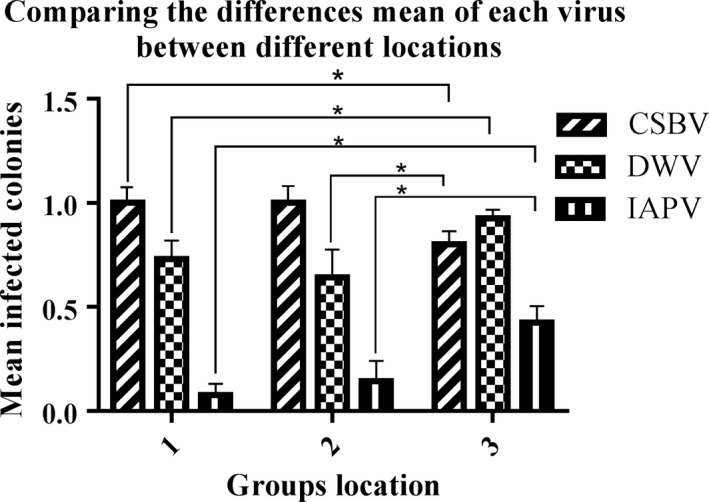
Comparing the means difference of each virus between different locations. 1, Fuan; 2, Cangshan; and 3, Yongtai group's location. Statistical analysis results were showed that there were significantly different (*p ≤ 0.05), in the distribution of the three viruses CSBV, Chinese sacbrood virus; deformed wing virus; DWV and Israeli acute paralysis virus; IAPV among different locations. Using ANOVA, Tukey HSD. Data are present the (mean with *SE*) of the test (*n* = 3), viruses, (*n *= 3) groups, 80 *Apis cerana cerana* colonies

**Figure 3:**
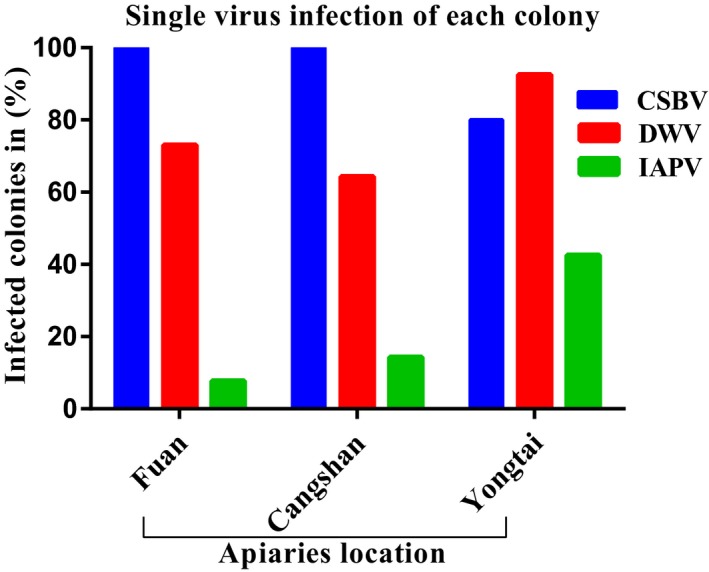
Prevalence of single‐virus infection of each *Apis cerana cerana* colony rate in (%), CSBV, Chinese sacbrood virus; DWV, deformed wing virus; and IAPV, Israeli acute paralysis virus. Based on the positive colonies (Fuan: *n *= 26, Cangshan: *n* = 14, and Yongtai: *n *= 40 colonies). Calculation: positive colonies ÷ total number of colonies in test *100 =  %

**Figure 4:**
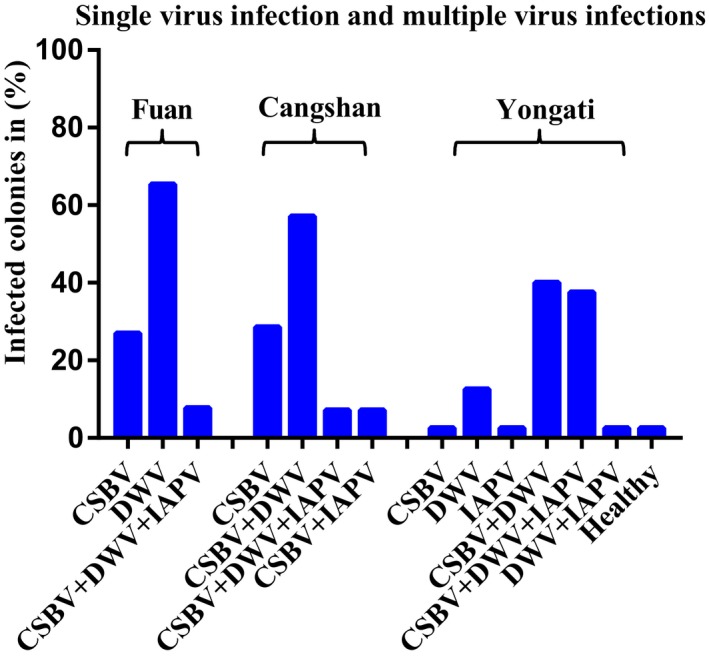
Percentage of single‐virus infection with multiple‐virus infection and no infection (healthy) each apiary for CSBV, Chinese sacbrood virus; DWV, deformed wing virus; and IAPV, Israeli acute paralysis virus. Based on the three locations of apiaries (Fuan: *n *= 26, Cangshan: *n* = 14, and Yongtai: *n* = 40 colonies) in the different locations in Fujian Province

A preliminary survey of 80 *A*. *c. cerana* colonies in the different locations of Fujian Province was performed to study the prevalence and distribution of seven honeybee viruses using the RT‐PCR method. In the present study, three types of honeybee viruses (CSBV, DWV, and IAPV) were detected in the larvae and pupae of *A*. *c. cerana* colonies from all the regions we sampled. However, CBPV, ABPV, KBV, and BQCV were not detected in any of the collected samples (Figures 2 and 3). Our results showed that the prevalence of CSBV was the maximum with detection in 90.0% of the samples tested, followed by that of DWV with detection in (81.25%) of the samples, whereas the lowest prevalence was observed for IAPV with detection in (26.25%) of the samples. Based on the apiaries, CSBV was the most prevalent in the Fuan apiary (100%) and in the Cangshan apiary (100%), DWV was the most prevalent in the Yongtai apiary (92.5%), and IAPV had the same prevalence in the Fuan and Cangshan apiaries. IAPV was the most prevalent in the Yongtai apiary (42.5%). In general, among the 80 *A*. *c. cerana* colonies, only one colony with (1.25%) prevalence was virus free, and this result is very interesting (Figure 3). Likewise, most of the colonies presented multiple‐virus infections, whereas a few colonies showed a single‐virus infection (Figure 4). During the sample collection, we observed *Varroa* mites in some colonies in the Yongtai apiary and symptoms of CSBV in the Fuan apiary; for that reason, those samples from both locations showed the highest prevalence, with (92.5%) DWV infection, but for CSBV in the Yongtai and Cangshan apiaries, most of the colonies did not present any disease symptoms and were very strong and healthy colonies, with eight to 10 combs per hive. Those colonies showed high infection rates so we can predict that it is possible that virus infection may be present in the colony without showing any obvious symptoms. In addition to viral infection in these different locations, we are aware of the relevance of *Varroa* mites, exposure, climate, malnutrition, mismanagement, bacteria, fungi, and other diseases that could be involved in the declining populations of *A*. *c. cerana* colonies in this province.

Comparing the distributions of viruses between different locations, data were analyzed using one‐way ANOVA, Post Hoc Test, Tukey HSD, with *p*‐value of ≤0.05 considered significant different. To compare the distribution of viruses across geographic locations, the viruses were placed in the dependent variable, and the locations were assigned as the grouping variable. Statistical analysis of the distribution results showed that there were significantly different (*p ≤ 0.05), in the distributions of the three viruses CSBV, DWV, and IAPV among different locations. (*n *= 3, viruses); *n* = 3 locations; 80 *A*. *c. cerana* colonies, *df* = 2; *F *= 4.813, 3.754, 6.213; *p* = 0.011, 0.028, and 0.003, respectively).

Comparing the distribution of CSBV, DWV, and IAPV among the Fuan and the Cangshan location showed no significant different (*p* = 1.000, 0.765, and 0.882, respectively), while comparing the distributions of CSBV and IAPV the Fuan and the Yongtai locations were significantly different (*p ≤ 0.05), but DWV was not significantly different (*p* = 0.112).

Furthermore comparing the distribution of CSBV and IAPV the Cangshan with the Yongtai location were not significantly different (*p* = 0.07, 0.091). However, the distribution of DWV did show significantly different (*p ≤ 0.05) (Figure 2).

## 4 DISCUSSION

China has the largest managed honeybee population in the world, with more than two million *A*. *c. cerana* and 8.77 million *A. mellifera* colonies. However, the presence of bee viruses is becoming a major problem, causing concern among beekeepers and economic losses. The prevalence of viral disease in various honeybee species has been investigated by several researchers, and it was observed that the trend of infection is worldwide (Shi et al., 2013; Wang et al., 2012; Xia, Mao, Wang, Zhou, & Wei, 2014).

Seven honeybee viruses of the two species, European honeybee (*A. mellifera*) and Chinese honeybee (*A*. *c. cerana*), were previously surveyed in China. DWV was the most widespread virus (94%) in *A. mellifera*, CSBV was a source of significant infection in *A*. *c. cerana* (86%), and the IAPV infection rate was (7%) (Ai et al., 2012). These results are similar to our findings that the prevalence of CSBV was (90%), followed by DWV at (81.25%), but our results for IAPV showed increasing infections at (26.25%) in *A*. *c. cerana* colonies. A few colonies were infected with a single virus, whereas most of the colonies were infected with multiple viruses. CSBV, IAPV, and DWV were detected in all apiaries. CSBV was the most prevalent in Fuan and Cangshan, DWV was the most prevalent in Yongtai and IAPV showed the same prevalence in Fuan and Cangshan, but IAPV was the most prevalent in Yongtai. However, BQCV, CBPV, ABPV, and KBV were not detected in any geographic locations (Figures 2 and 4).

The surveys of domestic *A*. *c. cerana* populations in China have suggested that CSBV is the primary disease involved in the declining population of colonies (Ai et al., 2012). The prevalence of CSBV in Taiwan increased from (41.1% to 69.6%), over 4 months in 2015. Additionally, (37.5%) of *A. mellifera* colonies were infected with CSBV, which showed that the western honeybee is also susceptible to CSBV (Nai et al., 2018). ABPV infection rate of (22%) in *A*. *c. cerana* was reported in Guangdong, while there was no infection of this virus in Fujian until now (Ding et al., 2016). IAPV detection was reported in Fujian Province with a prevalence of (11%) in *A. mellifera,* and the prevalence of IAPV in *A*. *c. cerana* was also reported in Hainan at (60%) and in Beijing at (30%–35%) (Antunez et al., 2012).

CSBV is a viral disease in the honeybee and is caused by SBV; first observed in Guangdong Province, China in 1972, it was named CSBV and is able to cause collapse of entire colonies (Hu et al., 2016). CSBV has existed in Fujian Province for nearly 50 years, causing death in a single colony and eventually the collapse of entire the colony (Xia, Zhou, & Wei, 2015). *A*. *c. cerana* is more susceptible to CSBV, whereas *A. mellifera* is less susceptible (Shan et al., 2017; Zhang, Zhang, Huang, & Han, 2014). CSBV has created a severe outbreak in *A*. *c. cerana* in China (Antunez et al., 2012). CSBV and SBV have similar genomes and sizes (26–30 nm in diameter), but they differ in their physiochemical characteristics, immunogenicity and pathogenicity in honeybees (Hu et al., 2016). CSBV belongs to the *Picornavirales* (order), *Iflaviridae* (family), and *Iflavirus* (genus) order, which represent a group of positive‐sense single‐stranded RNA viruses (Ahn et al., 2015; Hu et al., 2016; Reddy et al., 2017; Roy, Vidal‐Naquet, & Provost, 2016; Shan et al., 2017; Xia et al., 2015; Yoo et al., 2012). SBV infects mostly larvae, including adult bees, because the adult bees do not present disease symptoms and there is a failure of infection in pupae (Ahn et al., 2015; Choe, Nguyen, Hyun et al., 2012; Choe, Nguyen, Jin et al., 2012; Roy et al., 2016; Xia et al., 2014; Zhang et al., 2001). BQCV, ABPV, KBV, and IAPV belong to the *Dicistroviridae* (family) (Singh et al., 2010).

SBV has been detected throughout most of the world (Choe, Nguyen, Hyun et al., 2012; Choe, Nguyen, Jin et al., 2012; Hu et al., 2016). Infection of honeybees with SBV was reported from Myanmar, Nepal, and India, and more than (95%) of colonies collapsed due to Thailand sacbrood virus (TSBV). *A. cerana* in Korea has been threatened by SBV, which has resulted in severe colony loss (Yoo, Noh et al., 2012). The Korean honeybee *A. cerana* was infected with SBV with a prevalence of (60.49%) (Choe, Nguyen, Hyun et al., 2012; Choe, Nguyen, Jin et al., 2012). Many previous research works and reports have shown that SBV can cause outbreaks in both *A. cerana* and *A. mellifera* in different countries (Blanchard et al., 2014). In 2011, SBV (36%), DWV (100%), BQCV (93%), and CBPV (50%) infection prevalence was reported from Vietnam (Thu et al., 2016). SBV also infected *A. dorsata* (giant Asian honeybee), which was reported from Indonesia (Roberts & Anderson, 2014). The prevalence of SBV virus has been reported to be (97%) in France, DWV which has (100%) infected adult bee (Tantillo et al., 2015). The prevalence of SBV was reported in Denmark, France, Austria, India, Vietnam and China (Ai et al., 2012). The prevalence of SBV (18.33%), DWV (3.33%), and BQCV (6.11%) was reported from Vietnam (Thu et al., 2016).

There are two serotypes, a European serotype that infects *A. mellifera* throughout the world and an Asian serotype that can infect *A. cerana*, and multiple complete genomes and genetic variation of both serotypes have been described, including distinct strains in South Africa and Russia (Choe, Nguyen, Hyun et al., 2012; Choe, Nguyen, Jin et al., 2012; Roberts & Anderson, 2014). CSBV can infect honeybees at all stages from eggs to adults (Shan et al., 2017).

DWV is also the primary concern among the honeybee viruses that belongs to the *Iflaviridae,* (family) threatening honeybee colonies worldwide (Gisder & Genersch, 2017). KBV has not been detected in China in both species of honeybee until now (Ai et al., 2012). A survey of the honeybee *A. mellifera* in China from 2010 to 2013 showed losses of (10%) of colonies and overwintering losses of (770,000) colonies per year (Liu et al., 2016). While in 6 decades the number of honeybee colonies increased by (45%) globally, in recent decades, colony losses have increased, and overwintering losses have been reported frequently in different countries worldwide (Gisder & Genersch, 2015).

In conclusion, many studies have been conducted on the presence of honeybee viruses in western honeybees (*A. mellifera)* and Asian honeybees (*A. cerana*), but studies on Chinese honeybees (*A*. *c. cerana*) are still scarce. Molecular detection of bee viruses from this species is inadequate. Our recent report revealed the prevalence and distribution of seven bee viruses, especially the IAPV detection in *A*. *c. cerana* in the Fujian Province of China. This information could be used to investigate the spread of pathogens between and within bee populations in several regions of China. Besides, this study may help the beekeepers, control virus dispersal, epidemics and encourage the government to develop better strategies and breeding programs for honeybee resistance to viruses.

## CONFLICT OF INTERESTS

None declared.

## AUTHORS CONTRIBUTION

A. K. H., S. S. and S. H. conceived and designed the experiments, Z. L. contributed to the reagents and materials, A. K. H. and S. M. performed the experiments, M. R., M. F. R., and A. K. H. contributed to the sample collection, M. H. and A. K. H. wrote the manuscript with assistance from all authors, and M. Q. analyzed the data.

## ETHICS STATEMENT

None required.

## DATA ACCESIBILITY

All data are provided in the results section of this paper.

## References

[mbo3830-bib-0001] Ahn, A. J. , Ahn, K. S. , Suh, G. H. , Noh, J. H. , Kim, Y. H. , Yoo, M. S. , … Shin, S. S. (2015). Efficacy of silver ions against Sacbrood virus infection in the Eastern honey bee *Apis cerana* . Journal of Veterinary Science, 16, 3–289. 10.4142/jvs.2015.16.3.289 PMC4588014 25797295

[mbo3830-bib-0002] Ai, H. , Yan, X. , & Han, R. (2012). Occurrence and prevalence of seven bee viruses in *Apis mellifera* and *Apis cerana* apiaries in China. Journal of Invertebrate Pathology, 109, 160–164. 10.1016/j.jip.2011.10.006 22062807

[mbo3830-bib-0003] Antunez, K. , Anido, M. , Garrido‐Bailon, E. , Botias, C. , Zunino, P. , Martínez‐Salvador, A. , … Higes, M. (2012). Low prevalence of honeybee viruses in Spain during 2006 and 2007. Research in Veterinary Science, 93(3), 1441–1445. 10.1016/j.rvsc.2012.03.006 22513127

[mbo3830-bib-0004] Blanchard, P. , Guillot, S. , Antunez, K. , Koglberger, H. , Kryger, P. , de Miranda, J. R. , … Ribiere, M. (2014). Development and validation of a real‐time two‐step RT‐qPCR TaqMan assay for quantitation of Sacbrood virus (SBV) and its application to a field survey of symptomatic honey bee colonies. Journal of Virological Methods, 197, 7–13. 10.1016/j.jviromet.2013.09.012 24121133

[mbo3830-bib-0005] Chen, Y. P. , Higgins, J. A. , & Feldlaufer, M. F. (2005). Quantitative real‐time reverse transcription‐PCR analysis of deformed wing virus infection in the honeybee (*Apis mellifera* L.). Applied and Environmental Microbiology, 71, 436–441. 10.1128/AEM.71.1.436-441.2005 15640219 PMC544241

[mbo3830-bib-0006] Chen, Y. P. , Pettis, J. S. , Collins, A. , & Feldlaufer, M. F. (2006). Prevalence and transmission of honeybee viruses. Applied and environmental microbiology., 72, 606–611. 10.1128/AEM.72.1.606-611.2006 16391097 PMC1352288

[mbo3830-bib-0007] Choe, S. E. , Nguyen, T. T. , Hyun, B. H. , Noh, J. H. , Lee, H. S. , Lee, C. H. , & Kang, S. W. (2012). Genetic and phylogenetic analysis of South Korean sacbrood virus isolates from infected honey bees (*Apis cerana*). Veterinary Microbiology, 157, 32–40. 10.1016/j.vetmic.2011.12.007 22221381

[mbo3830-bib-0008] Choe, S. E. , Nguyen, L. T. K. , Jin, H. N. , Hong, B. K. , Jean, Y. H. , Chang, H. K. , & Kang, S. W. (2012). Prevalence and distribution of six bee viruses in Korean *Apis cerana* populations. Journal of Invertebrate Pathology, 109, 330–333. 10.1016/j.jip.2012.01.003 22273697

[mbo3830-bib-0009] Ding, G. , Fondevila, N. , Palacio, M. A. , Merke, J. , Martinez, A. , Camacho, B. , … Shi, W. (2016). Prevalence of honeybee viruses in different regions of China and Argentina. Revue Scientifique et Technique, 35, 825–833. 10.20506/rst.35.3.2572 28332647

[mbo3830-bib-0010] Galbraith, D. A. , Fuller, Z. L. , Ray, A. M. , Brockmann, A. , Frazier, M. , Gikungu, M. W. , … Kocher, S. D. (2018). Investigating the viral ecology of global bee communities with high‐throughput metagenomics. Scientific Reports, 8, 1–11. 29891995 10.1038/s41598-018-27164-z PMC5995813

[mbo3830-bib-0011] Gisder, S. , & Genersch, E. (2015). Special issue: Honey bee viruses. Viruses, 7, 5603–5608. 26702462 10.3390/v7102885 PMC4632393

[mbo3830-bib-0012] Gisder, S. , & Genersch, E. (2017). Viruses of commercialized insect pollinators. Journal of Invertebrate Pathology, 147, 51–59. 10.1016/j.jip.2016.07.010 27498219

[mbo3830-bib-0013] Hu, Y. , Fei, D. , Jiang, L. , Wei, D. , Li, F. , Diao, Q. , & Ma, M. (2016). A comparison of biological characteristics of three strains of Chinese sacbrood virus in *Apis cerana* . Scientific Reports, 6, 1–14. 27853294 10.1038/srep37424 PMC5112594

[mbo3830-bib-0014] Huang, W. F. , Mehmood, S. , Huang, S. , Chen, Y. W. , Ko, C. Y. , & Su, S. (2017). Phylogenetic analysis and survey of *Apis cerana* strain of Sacbrood virus (AcSBV) in Taiwan suggests a recent introduction. Journal of Invertebrate Pathology, 146, 36–40. 10.1016/j.jip.2017.04.001 28390783

[mbo3830-bib-0015] Liu, Z. , Chen, C. , Niu, Q. , Qi, W. , Yuan, C. , Su, S. , … Shi, W. (2016). Survey results of honey bee (*Apis mellifera*) colony losses in China (2010–2013). Journal of Apicultural Research, 55, 1–8. 10.1080/00218839.2016.1193375

[mbo3830-bib-0016] Martinello, M. , Baratto, C. , Manzinello, C. , Piva, E. , Borin, A. , Toson, M. , … Mutinelli, F. (2017). Spring mortality in honey bees in northeastern Italy: detection of pesticides and viruses in dead honey bees and other matrices. Journal of Apicultural Research, 56, 239–254. 10.1080/00218839.2017.1304878

[mbo3830-bib-0017] McMenamin, A. J. , & Flenniken, M. L. (2018). Recently identified bee viruses and their impact on bee pollinators. Current Opinion in Insect Science, 26, 120–129. 10.1016/j.cois.2018.02.009 29764651

[mbo3830-bib-0018] Nai, Y.‐S. , Ko, C.‐Y. , Hsu, P.‐S. , Tsai, W.‐S. , Chen, Y.‐W. , Hsu, M.‐H. , & Sung, I. H. (2018). The seasonal detection of AcSBV (*Apis cerana* sacbrood virus) prevalence in Taiwan. Journal of Asia‐Pacific Entomology, 21, 417–422. 10.1016/j.aspen.2018.02.003

[mbo3830-bib-0019] Reddy, K. E. , Thu, H. T. , Yoo, M. S. , Ramya, M. , Reddy, B. A. , Lien, N. T. K. , … Quyen, D. V. (2017). Comparative genomic analysis for genetic variation in sacbrood virus of *Apis cerana* and *Apis mellifera* honeybees from different regions of Vietnam. Journal of Insect Science, 50, 1–10. 10.1093/jisesa/iex077 PMC5634237 29117376

[mbo3830-bib-0020] Roberts, J. M. , & Anderson, D. L. (2014). A novel strain of sacbrood virus of interest to world apiculture. Journal of Invertebrate Pathology, 118, 71–74. 10.1016/j.jip.2014.03.001 24650855

[mbo3830-bib-0021] Roy, C. , Vidal‐Naquet, N. , & Provost, B. (2016). A severe sacbrood virus outbreak in a honeybee (*Apis mellifera* L.) colony: a case report. Veterinární Medicína, 60, 330–335. 10.17221/VETMED

[mbo3830-bib-0022] Schoonvaere, K. , Smagghe, G. , Francis, F. , & de Graaf, D. C. (2018). Study of the metatranscriptome of eight social and solitary wild bee species reveals novel viruses and bee parasites. Frontiers in Microbiology, 9, 177 10.3389/fmicb.2018.00177 29491849 PMC5817871

[mbo3830-bib-0023] Shan, L. , Liuhao, W. , Jun, G. , Yujie, T. , Yanping, C. , Jie, W. , & Jilian, L. (2017). Chinese Sacbrood virus infection in Asian honey bees (*Apis cerana cerana*) and host immune responses to the virus infection. Journal of Invertebrate Pathology, 150, 63–69. 10.1016/j.jip.2017.09.006 28916146

[mbo3830-bib-0024] Shi, Y. Y. , Sun, L. X. , Huang, Z. Y. , Wu, X. B. , Zhu, Y. Q. , Zheng, H. J. , & Zeng, Z. J. (2013). A SNP based high‐density linkage map of *Apis cerana* reveals a high recombination rate similar to *Apis mellifera* . PLoS ONE, 8, 1–6. 10.1371/journal.pone.0076459 PMC3794977 24130775

[mbo3830-bib-0025] Singh, R. , Levitt, A. L. , Rajotte, E. G. , Holmes, E. C. , Ostiguy, N. , Vanengelsdorp, D. , … Cox‐Foster, D. L. (2010). RNA viruses in hymenopteran pollinators: evidence of inter‐Taxa virus transmission via pollen and potential impact on non‐*Apis* hymenopteran species. PLoS ONE, 5, 1–16. 10.1371/journal.pone.0014357 PMC3008715 21203504

[mbo3830-bib-0026] Tantillo, G. , Bottaro, M. , di Pinto, A. , Martella, V. , di Pinto, P. , & Terio, V. (2015). Virus infections of honeybees *Apis mellifera* . The Italian Journal of Food Safety, 4, 157–168. 10.4081/ijfs.2015.5364 PMC5076640 27800411

[mbo3830-bib-0027] Thu, H. T. , Thi Kim Lien, N. , Thuy Linh, M. , Le, T. H. , Hoa, N. T. , Hong Thai, P. , … Kang, S. W . (2016). Prevalence of bee viruses among *Apis cerana* populations in Vietnam. Journal of Apicultural Research, 55(5), 379–385. 10.1080/00218839.2016.1251193

[mbo3830-bib-0028] Wang, Z. L. , Liu, T. T. , Huang, Z. Y. , Wu, X. B. , Yan, W. Y. , & Zeng, Z. J. (2012). Transcriptome analysis of the Asian honey bee *Apis cerana cerana* . PLoS ONE, 7, 7–11. 10.1371/journal.pone.0047954 PMC3480438 23112877

[mbo3830-bib-0029] Ward, L. , Waite, R. , Boonham, N. , Fisher, T. , Pescod, K. , Thompson, H. , … Brown, M. (2007). First detection of Kashmir bee virus in the UK using real‐time PCR. Apidologie, 38, 181–190. 10.1051/apido:2006072

[mbo3830-bib-0030] Xia, X. , Mao, Q. , Wang, H. , Zhou, B. , & Wei, T. (2014). Replication of Chinese sacbrood virus in primary cell cultures of Asian honeybee (*Apis cerana*). Archives of Virology, 159, 3435–3438. 10.1007/s00705-014-2183-3 25139546

[mbo3830-bib-0031] Xia, X. , Zhou, B. , & Wei, T. (2015). Complete genome of Chinese sacbrood virus from *Apis cerana* and analysis of the 3C‐like cysteine protease. Virus Genes, 50, 277–285. 10.1007/s11262-014-1154-9 25557929

[mbo3830-bib-0032] Yang, B. , Peng, G. , Li, T. , & Kadowaki, T. (2013). Molecular and phylogenetic characterization of honey bee viruses, Nosema microsporidia, protozoan parasites, and parasitic mites in China. Ecology and Evolution, 3, 298–311. 10.1002/ece3.464 23467539 PMC3586640

[mbo3830-bib-0033] Yoo, M. S. , Noh, J. H. , Yoon, B. S. , Reddy, K. E. , Kweon, C. H. , Jung, S. C. , & Kang, S. W. (2012). Reverse transcription loop‐mediated isothermal amplification for sensitive and rapid detection of Korean sacbrood virus. Journal of Virological Methods, 186, 147–151. 10.1016/j.jviromet.2012.08.009 22947691

[mbo3830-bib-0034] Yoo, M. S. , Thi, K. C. , van Nguyen, P. , Han, S. H. , Kwon, S. H. , & Yoon, B. S. (2012). Rapid detection of sacbrood virus in honeybee using ultra‐rapid real‐time polymerase chain reaction. Journal of Virological Methods, 179, 195–200. 10.1016/j.jviromet.2011.10.014 22079620

[mbo3830-bib-0035] Zhang, J. , Feng, J. , Liang, Y. , Chen, D. , Zhou, Z. H. , Zhang, Q. , & Lu, X. (2001). Three‐dimensional structure of the Chinese Sacbrood bee virus. Science China Life Sciences, 44, 443–448. 10.1007/BF02879612 18726426

[mbo3830-bib-0036] Zhang, Y. , Huang, X. , Xu, Z. , Han, R. , & Chen, J. (2013). Differential gene transcription in honeybee (*Apis cerana*) larvae challenged by Chinese Sacbrood Virus (CSBV). Sociobiology, 60, 413–420.

[mbo3830-bib-0037] Zhang, Y. , Zhang, G. , Huang, X. , & Han, R. (2014). Proteomic analysis of *Apis cerana* and *Apis mellifera* larvae fed with heterospecific royal jelly and by CSBV challenge. PLoS ONE, 9, 1–19. 10.1371/journal.pone.0102663 PMC4125304 25102167

